# Sevoflurane upregulates neuron death process-related Ddit4 expression by NMDAR in the hippocampus

**DOI:** 10.18632/aging.204822

**Published:** 2023-06-21

**Authors:** Shuai Li, Qi Hou, Runjia Wang, Yu Hou, Qiang Wang, Bo Zhang, Cheng Ni, Hui Zheng

**Affiliations:** 1 Department of Anesthesiology, National Cancer Center/National Clinical Research Center for Cancer/Cancer Hospital, Chinese Academy of Medical Sciences and Peking Union Medical College, Beijing 100021, China

**Keywords:** sevoflurane, neuronal death process, Ddit4, NMDAR

## Abstract

Postoperative cognitive dysfunction (POCD) is a serious and common complication induced by anesthesia and surgery. Neuronal apoptosis induced by general anesthetic neurotoxicity is a high-risk factor. However, a comprehensive analysis of general anesthesia-regulated gene expression patterns and further research on molecular mechanisms are lacking. Here, we performed bioinformatics analysis of gene expression in the hippocampus of aged rats that received sevoflurane anesthesia in GSE139220 from the GEO database, found a total of 226 differentially expressed genes (DEGs) and investigated hub genes according to the number of biological processes in which the genes were enriched and performed screening by 12 algorithms with cytoHubba in Cytoscape. Among the screened hub genes, *Agt*, *Cdkn1a*, *Ddit4*, and *Rhob* are related to the neuronal death process. We further confirmed that these genes, especially *Ddit4*, were upregulated in the hippocampus of aged mice that received sevoflurane anesthesia. *NMDAR*, the core target receptor of sevoflurane, rather than *GABA_A_R*, mediates the sevoflurane regulation of *DDIT4* expression. Our study screened sevoflurane-regulated DEGs and focused on the neuronal death process to reveal *DDIT4* as a potential target mediated by *NMDAR*, which may provide a new target for the treatment of sevoflurane neurotoxicity.

## INTRODUCTION

More than 300 million operations are performed worldwide each year, and a continued increase is observed in all economic environments [1]. Postoperative cognitive dysfunction (POCD) is a common cognitive impairment in patients during the perioperative period and is mostly found in elderly patients, with a reported incidence ranging from 15% to 60% [2, 3]. POCD is mainly characterized by progressive postoperative memory impairment, cognitive decline, and executive dysfunction. In addition, the effects of POCD may not be temporary and can lead to neurological dysfunction years after surgery [4, 5], which is associated with an increased risk of life-threatening illness and death. Neuronal apoptosis, a high-risk factor inducing POCD, leads to decreased neurogenesis, impaired synaptic plasticity, neuroinflammation, and oxidative stress in POCD patients [6–8]. Sevoflurane can induce POCD-related behaviors in animal, such as mice [9] or rats [10, 11]. However, the neurobiological basis of sevoflurane neurotoxicity remains largely unknown.

General anesthetic neurotoxicity has been extensively examined in recent years [12–14]. An increasing number of studies have shown that inhaled anesthetics may cause neurotoxicity, leading to hippocampal neuronal damage and apoptosis, which result in cognitive dysfunction [15–17]. Sevoflurane, the most commonly used inhalation anesthetic, induces neuronal apoptosis [18–22]. Sevoflurane enhanced the production of lactate in aged marmoset brains [23]. Lactate accumulation can induce neuronal apoptosis or even acidosis in critically ill patients [24–26]. Sevoflurane was shown to activate gamma-aminobutyric acid subtype A receptor (*GABA_A_R*) to induce apoptosis of immature dentate granule cells in mice [27]. Apoptosis is regulated by multiple pathways, among which the mechanism of neuronal apoptosis induced by sevoflurane through related signaling pathways has attracted increased attention [28–30]. Sevoflurane inhibits the *ERK1/2* signaling pathway by antagonizing the N-methyl-D-aspartate receptor (*NMDAR*) and upregulates the expression of the apoptotic proteins caspase-3 and *Bax* in mitochondria, resulting in apoptosis of hippocampal neurons [31]. Additionally, sevoflurane promotes the expression of the apoptotic factor connexin 43 (*Cx43*) and leads to neuronal apoptosis by activating the *JNK/cJun/AP-1* signaling pathway [32]. However, a comprehensive analysis of the differentially expressed genes (DEGs) regulated by sevoflurane and further investigation of the molecular mechanism is lacking.

Transcriptomic analysis has identified comprehensive gene expression patterns to help reveal potential mechanisms for various neurological diseases [33, 34] and has shown that inhaled anesthetic are associated with neurological damage [35]. Here, we applied multistage comprehensive bioinformatics methods to explore the possible pathogenesis of sevoflurane neurotoxicity. We focused on the potential key genes with different expression levels in the hippocampus of aged rats after sevoflurane anesthesia, established the functional annotation of their potential target genes and used gene enrichment analysis to reveal the role of DEGs associated with the cell death process in sevoflurane anesthesia. We further performed an *in vivo* experiment in aged mice that received sevoflurane exposure to confirm the pattern of upregulation of these genes in the hippocampus and further found that the *NMDAR* mediates the sevoflurane regulation of *DDIT4* expression. This work showed the molecular mechanism of sevoflurane-induced neuronal apoptosis and provided a new potential target for sevoflurane toxicity.

## RESULTS

### DEG identification

**Figure 1:**
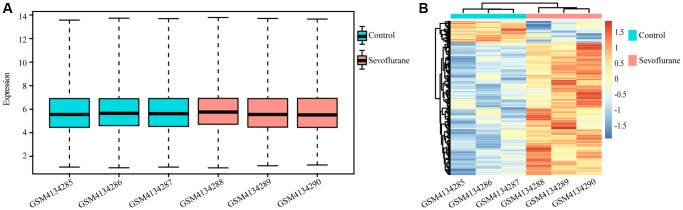
**Differentially regulated genes between the control and sevoflurane-treated groups.** (**A**) Boxplot of all genes in each sample. (**B**) Heatmap of differentially regulated genes in the control and sevoflurane-treated groups, 194 upregulated and 32 downregulated genes after treatment with 2.5% sevoflurane in 100% oxygen for 4 hours. (Green: Control group; Red: sevoflurane-treated group).

A total of 10032 unique genes were annotated with the SwissProt database. The expression boxplot of all genes for each sample is shown in Figure 1A after normalization with the rma function by using the oligo package. The differential expression analysis identified 194 upregulated and 32 downregulated genes after treatment with 2.5% sevoflurane in 100% oxygen for 4 hours in an anesthetizing chamber with the criterion of a *P* value less than 0.05. Among all DEGs, 160 DEGs (153 upregulated DEGs and 17 downregulated DEGs) could be annotated in metascape, they were listed in Table 1, and the heatmap of the DEGs between the two groups is displayed in Figure 1B.

**Table 1:** Differentially regulated genes between the control and sevoflurane-treated groups.

	**Differentially regulated genes**
153 Upregulated genes in the sevoflurane-treated group.	*Slc19a3; Tmem163; Snx9; Ptp4a3; Rabif; Rnf125; Creg1; Dbt; Ccdc115; Bcas2; Abhd15; Lsm5; Olfr1283; Pex10; Olfr1499; Olfr273; Timm29; Lonrf3; Map3k6; Pla2g3; Tfcp2l1; Olfr325; Olfr432; Serpinb3a; Krtap13; Hddc2; BC005624; Olfr362; Hipk2; Slc2a12; Olfr1406; Acer2; Arrdc2; Zfp521; Peli2; Sun2; Mocs1; Parvg; Ctxn2; Slc2a9; Cldn2; Srp9; Ecrg4; Sf3b5; Ikzf2; Ikzf1; Top3b; Mlc1; Fam83d; Trim21; Pdcd5; Mrgprb3; Gm266; Olfr1321; Tnfrsf11a; Dlc1; Lrig3; Sult1d1; Elmo1; Olfr1451; Olfr1299; H1f3; Tceal5; Ddah1; Fxyd1; Gpd1; Smarcd2; Nfe2l2; Pex11a; Ackr3; Agt; C3; Pla2g1b; Gsta3; A2m; Mag; Aldoc; Gpr139; Sparc; Plat; Pdgfra; Ptgds; Tgfbr3; Oprm1; Rpl36; Aqp4; Tmbim6; Mertk; Phactr2; Rhob; Hrk; Pcp4; Timp4; Hand1; Klf9; Cntfr; Afg1l; Adipor2; Fam43a; Rgs16; Ehd2; Spint1; Rassf4; Prr5; Gramd3; Wdr89; Klra8; Lims2; Cnksr3; Rac2; Fkbp14; Ech1; Plcd4; Acsl3; Gbp2; Prlhr; Cdkn1a; Prkg2; Prkch; Psat1; Etfb; Tmem252; Gsto2; Prxl2a; Paqr8; Rab31; Usp54; Clic1; Bspry; Cnppd1; Slc10a6; Slc3a2; Zfp24; Slc29a3; Rcan2; Ddit4; Dhrs4; Cygb; Igsf1; Plce1; Tsc22d3; Zfp422; Slc38a2; Plcb4; Slc12a2; Pip4k2a; Apln; Olig1; Timm8a1; Ppm1f; Rapgef3*
17 Downregulated genes in the sevoflurane-treated group.	*Olfr1461; Flrt2; Kif21b; Olfr1474; Kcnh7; Egr1; Hbb-bs; Nr4a3; Kcnv1; Gpam; H2aw; Itm2a; Slc16a4; Hbb-bs; Hba-a1; Prom1; Tspan2.*

### Functional enrichment analysis of DEGs

**Figure 2:**
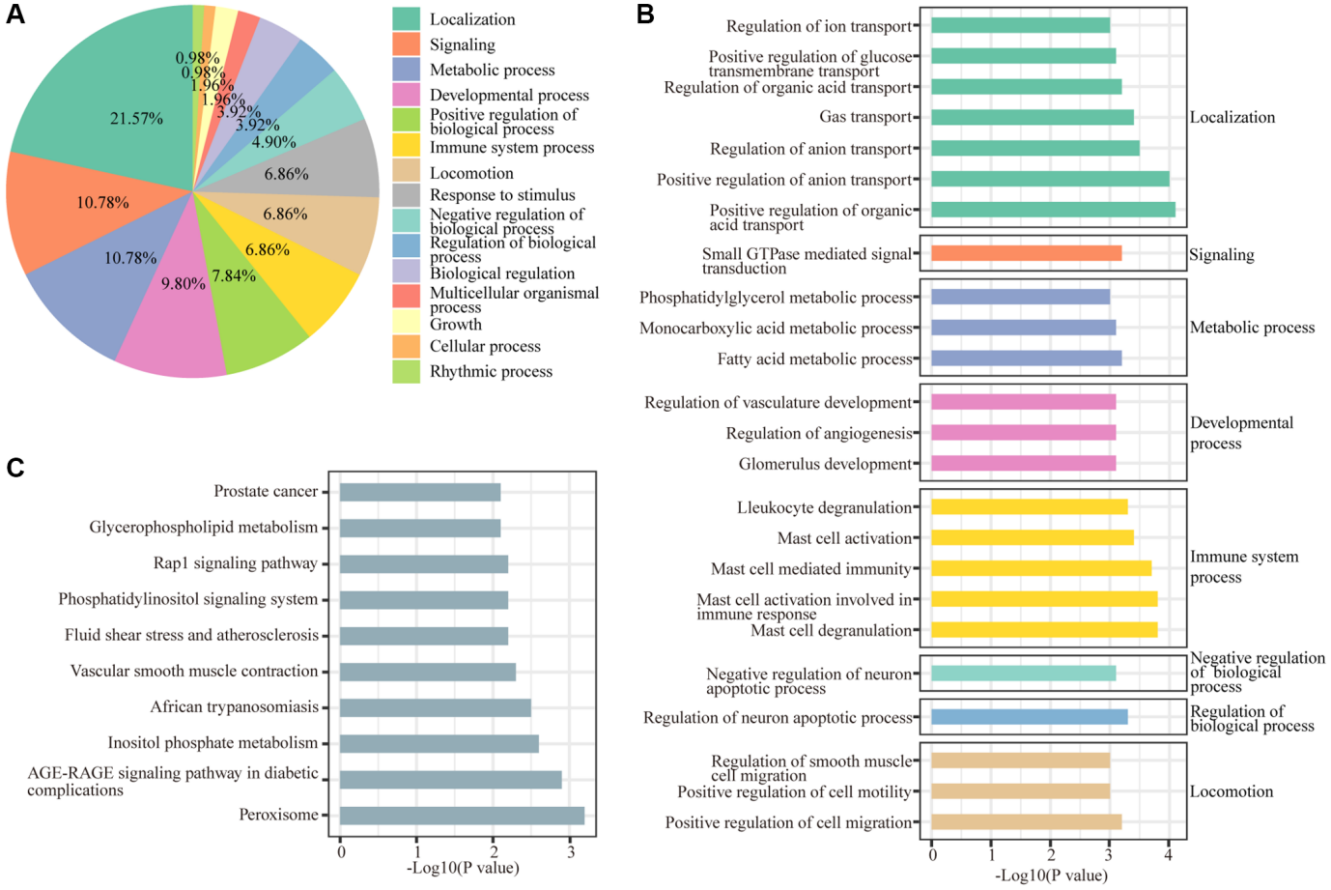
**GO and KEGG enrichment analyses of differentially regulated genes.** (**A**) Functions of biological processes that are significantly enriched by differentially expressed genes. (**B**) Top 20 significantly enriched biological processes. (**C**) Ten KEGG pathways were significantly enriched by differentially expressed genes.

**Figure 3:**
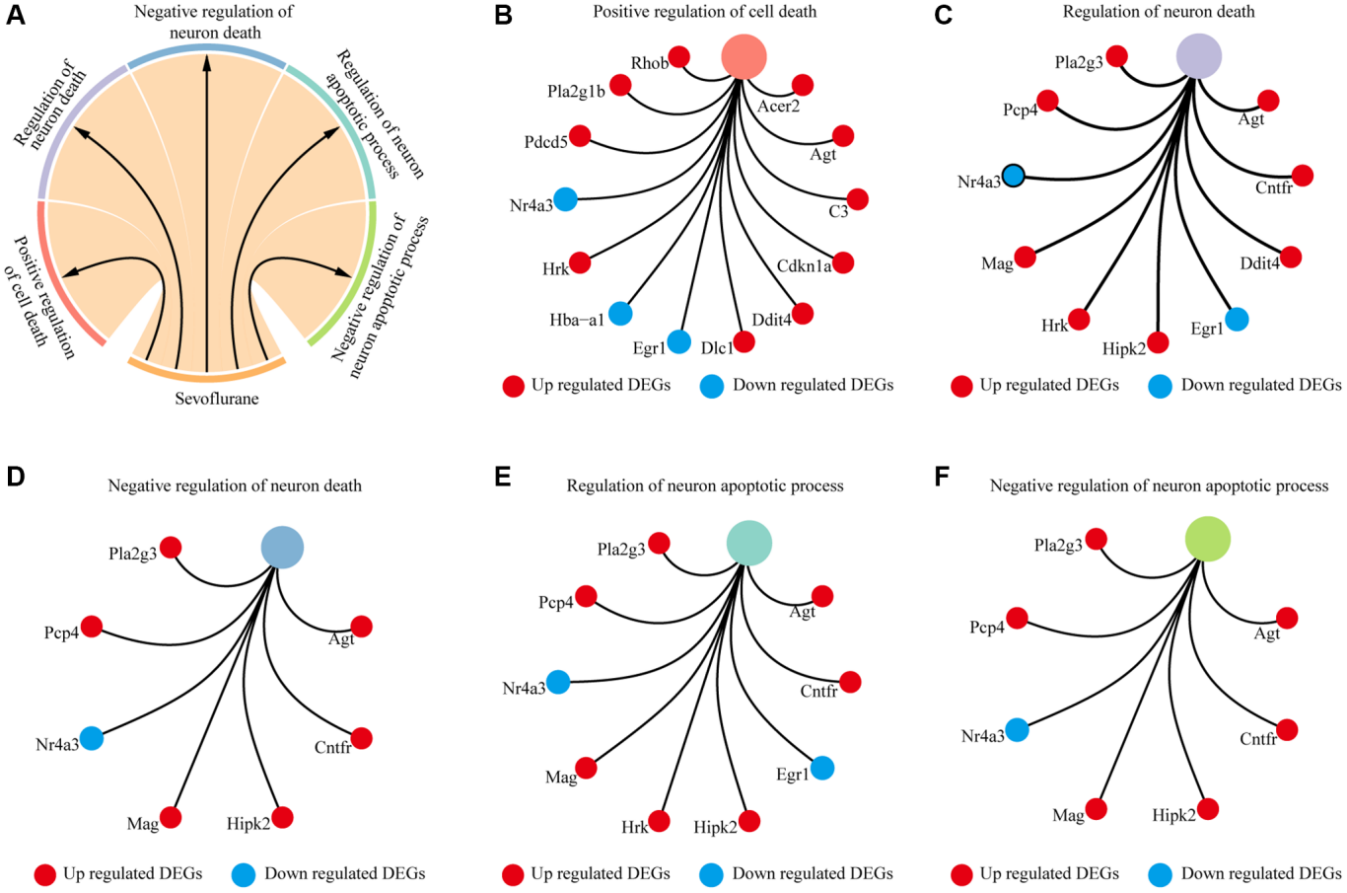
**Cell death-related biological processes were significantly enriched by differentially expressed genes.** (**A**) Five cell death-related biological processes were significantly enriched by differentially expressed genes. (**B**) Thirteen differentially expressed genes (10 genes upregulated in the sevoflurane-treated group and 3 genes downregulated in the sevoflurane-treated group) were significantly enriched in positive regulation of cell death. (**C**) Ten differentially expressed genes (8 genes upregulated in the sevoflurane-treated group and 2 genes downregulated in the sevoflurane-treated group) were significantly enriched in the regulation of neuronal death. (**D**) Seven differentially expressed genes (6 genes upregulated in the sevoflurane-treated group and 1 gene downregulated in the sevoflurane-treated group) were significantly enriched in the negative regulation of neuronal death. (**E**) Nine differentially expressed genes (7 genes upregulated in the sevoflurane-treated group and 2 genes downregulated in the sevoflurane-treated group) were significantly enriched in the regulation of neuronal apoptosis. (**F**) Seven differentially expressed genes (6 genes upregulated in the sevoflurane-treated group and 1 gene downregulated in the sevoflurane-treated group) were significantly enriched in the negative regulation of neuronal apoptosis. (Red: up regulated DEGs; blue: down regulated DEGs).

Biological processes and KEGG annotation were applied to explore the function of DEGs. All DEGs significantly played a role in localization, signaling, metabolic process, development process, and positive regulation of biological process (Figure 2A). Twenty-four biological process terms were filtered with *P* value less than 0.001, and DEGs were significantly enriched in the regulation of neuronal apoptosis (Figure 2B). Next, we screened the biological processes associated with neuron death with the key words neuron and death, and 5 biological processes (positive regulation of cell death, regulation of neuron death, negative regulation of neuron death, regulation of neuron apoptotic process and negative regulation of neuron apoptotic process) were dysregulated by sevoflurane (Figure 3A). Most of the genes enriched in disordered biological processes associated with cell death were upregulated after sevoflurane inhalation (Figure 3B–3F). A total of 10 KEGG pathways, such as peroxisome, AGE-RAGE signaling pathway in diabetic complications, inositol phosphate metabolism, vascular smooth muscle contraction, rap1 signaling pathway, and glycerophospholipid metabolism, were enriched by DEGs (Figure 2C).

### Protein-protein interaction network construction and hub gene selection

**Figure 4:**
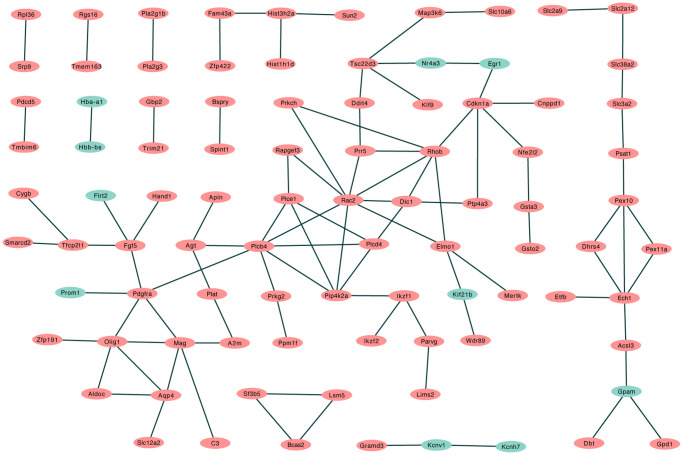
**PPI network of differentially regulated genes in STRING.** (Red: upregulated differentially expressed genes in the sevoflurane-treated group; green: downregulated differentially expressed genes in the sevoflurane-treated group).

**Figure 5:**
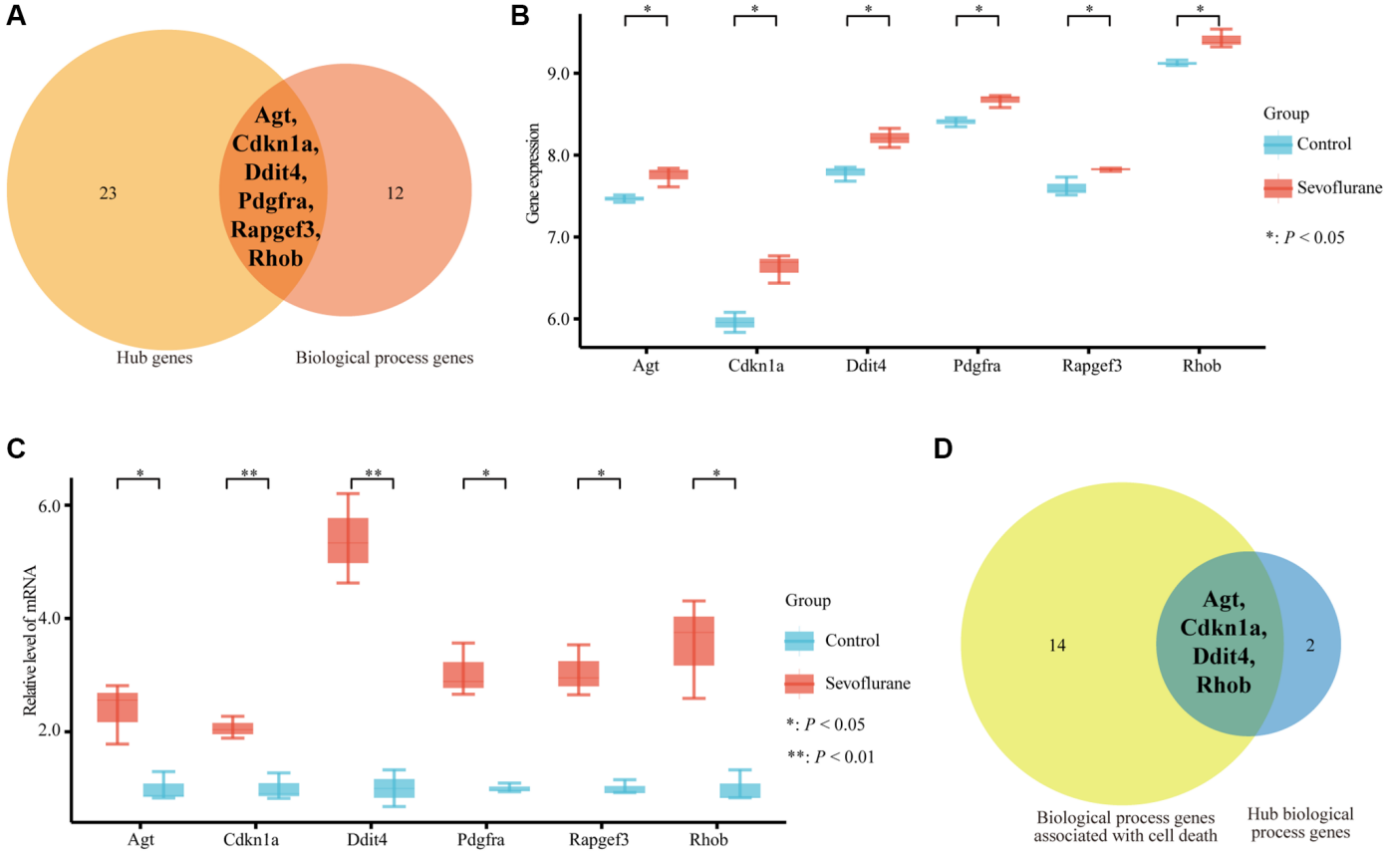
**Six hub genes were enriched in biological process terms, and 4 hub genes were associated with cell death biological process terms.** (**A**) Six hub genes enriched in biological process terms. (**B**) Expression of 6 hub genes between the control and sevoflurane-treated groups. (**C**) qPCR detection of *Agt*, *Cdkn1a*, *Ddit4*, *Pdgfra*, *Rapgef3*, and *Rhob* mRNA expression levels in the hippocampus of the mice that received 3% sevoflurane exposure for 4 h or the control mice. Sevoflurane indicates the mice received sevoflurane exposure. Control indicates that the mice were raised only under normal conditions. (**D**) Four hub genes associated with cell death biological process terms. The data shown are the means ± SDs, *n* = 3. ^*^P < 0.05, ^**^P < 0.01. (Green: Control group; Red: sevoflurane-treated group).

A total of 57 nodes and 97 interactions of the DEGs were identified in STRING and were visualized in Cytoscape (Figure 4). We calculated the number of genes enriched in biological process terms, and the genes that were enriched in at least 10 terms are listed in Table 2. The cytoHubba application identified 58 hub genes with 12 algorithms, including 29 genes that were identified by at least five different methods as candidate hub genes (Table 3). Six hub genes (*Agt*, *Cdkn1a*, *Ddit4*, *Pdgfra*, *Rapgef3*, and *Rhob*) were both selected with two methods (Figure 5A). The six hub genes were upregulated after sevoflurane inhalation (Figure 5B). We further validated the expression of the six hub genes *in vivo.* We found that 4 h of 3% sevoflurane treatment increased the mRNA levels of *Agt*, *Cdkn1a*, *Ddit4*, *Pdgfra*, *Rapgef3*, and *Rhob* in the mouse hippocampus (Figure 5C). Among the 6 hub genes, 4 genes (*Agt*, *Cdkn1a*, *Ddit4,* and *Rhob*) were also enriched in biological processes associated with neuronal death (Figure 5D).

**Table 2:** Number of biological process terms per gene involved.

**Genes**	**Terms**	**Genes**	**Terms**	**Genes**	**Terms**	**Genes**	**Terms**	**Genes**	**Terms**
*Agt*	57	*Pla2g1b*	11	*Prr5*	6	*Mag*	4	*Ech1*	2
*Nr4a3*	40	*Gpam*	10	*Rac2*	6	*Rab31*	4	*Etfb*	2
*Pla2g3*	34	*Sparc*	10	*Cnksr3*	5	*Serpinb3a*	4	*Kcnh7*	2
*Pdgfra*	27	*Tgfbr3*	10	*Cntfr*	5	*Slc10a6*	4	*Kcnv1*	2
*Egr1*	25	*Dlc1*	9	*Fgf5*	5	*Slc16a4*	4	*Klf9*	2
*Nfe2l2*	22	*Plce1*	9	*Flrt2*	5	*Sun2*	4	*Slc2a9*	2
*Rapgef3*	21	*Ackr3*	8	*Mertk*	5	*Aqp4*	3	*Trim21*	2
*Hipk2*	20	*Fxyd1*	8	*Oprm1*	5	*Clic1*	3	*Aldoc*	1
*C3*	17	*Pcp4*	8	*Plat*	5	*Cygb*	3	*Cnppd1*	1
*Rhob*	16	*Tnfrsf11a*	8	*Prkg2*	5	*Ecrg4*	3	*Elmo1*	1
*Ptgds*	14	*Acsl3*	7	*Snx9*	5	*Hand1*	3	*Gm266*	1
*Ppm1f*	13	*Apln*	7	*Timp4*	5	*Pip4k2a*	3	*Lims2*	1
*Slc12a2*	13	*Ddah1*	7	*Tmbim6*	5	*Plcb4*	3	*Paqr8*	1
*Ddit4*	12	*Pdcd5*	7	*A2m*	4	*Plcd4*	3	*Peli2*	1
*Adipor2*	11	*Spint1*	7	*Hba-a1*	4	*Prom1*	3	*Prlhr*	1
*Cdkn1a*	11	*Acer2*	6	*Hrk*	4	*Slc38a2*	3	*Rabif*	1
*Mrgprb3*	11	*Pla2g1b*	11	*Ikzf1*	4	*Slc3a2*	3		

**Table 3:** Times of hub genes selected from 12 algorithms with cytoHubba in cytoscape.

**Genes**	**Times**	**Genes**	**Times**	**Genes**	**Times**	**Genes**	**Times**	**Genes**	**Times**
*Ddit4*	12	*Plcd4*	10	*Tsc22d3*	8	*A2m*	3	*Pla2g3*	3
*Elmo1*	12	*Plce1*	10	*Prkch*	7	*Bcas2*	3	*Prom1*	3
*Mag*	12	*Agt*	9	*Kcnv1*	6	*Cygb*	3	*Psat1*	3
*Olig1*	12	*Cdkn1a*	9	*Kif21b*	6	*Dbt*	3	*Sf3b5*	3
*Pdgfra*	12	*Fgf5*	9	*Ptp4a3*	6	*Gpam*	3	*Apln*	2
*Ddit4*	12	*Ikzf1*	9	*Rapgef3*	6	*Gsto2*	3	*Map3k6*	2
*Pip4k2a*	12	*Prkg2*	9	*Dhrs4*	5	*Hist3h2a*	3	*Ppm1f*	2
*Plcb4*	12	*Tfcp2l1*	9	*Egr1*	5	*Lsm5*	3	*Slc3a2*	2
*Prr5*	12	*Ech1*	8	*Acsl3*	4	*Mertk*	3	*Ikzf2*	1
*Rac2*	12	*Gsta3*	8	*Aldoc*	4	*Parvg*	3	*Slc38a2*	1
*Rhob*	12	*Pex10*	8	*Dlc1*	4	*Pex11a*	3	*Wdr89*	1
*Aqp4*	10	*Plat*	8	*Nfe2l2*	4	*Pla2g1b*	3		

### Sevoflurane upregulated the expression of *Ddit4*

**Figure 6:**
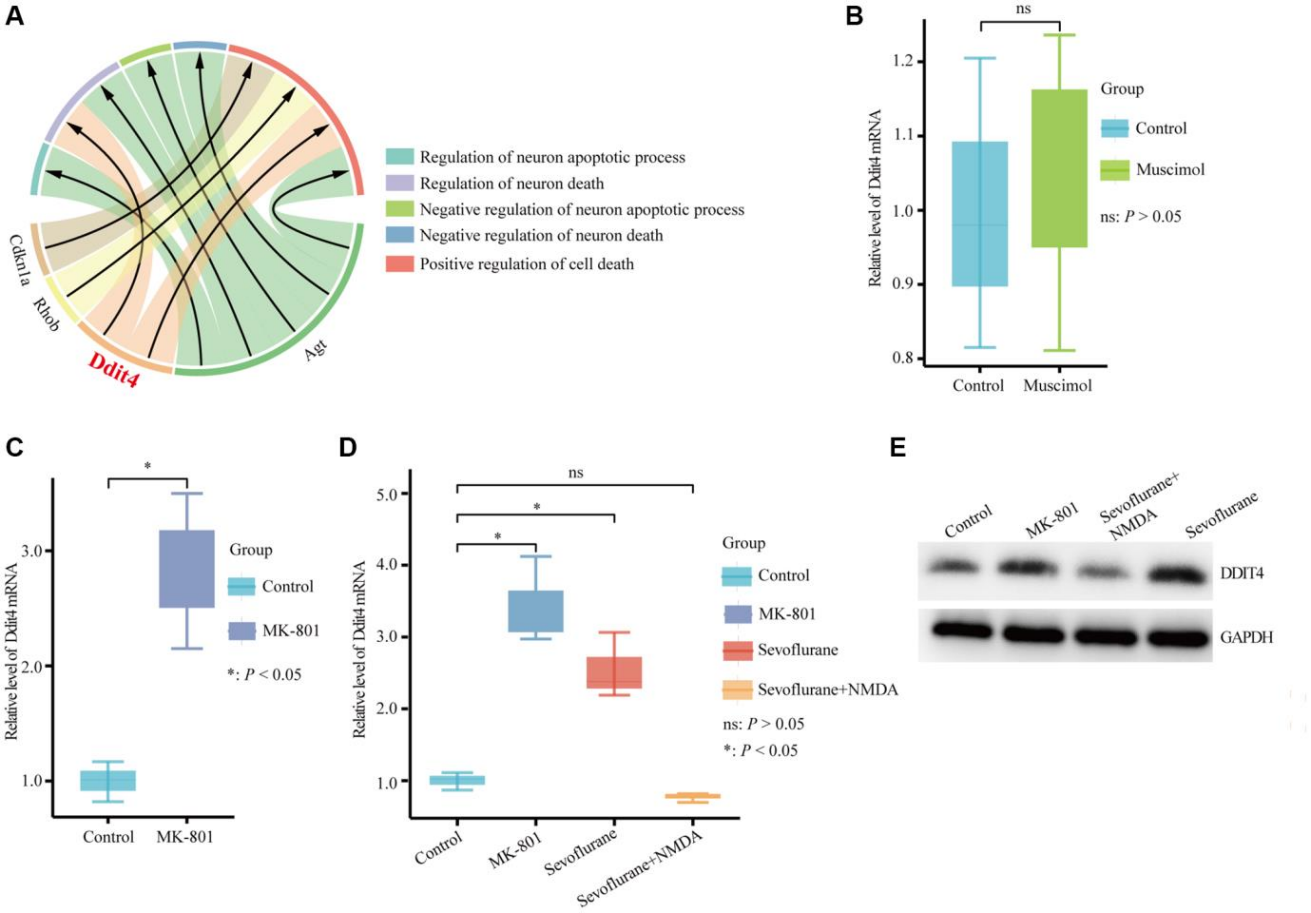
**Hub genes associated with cell death biological process terms.** (**A**) Four hub genes enriched in cell death biological process terms. (**B**) qPCR detection of the Ddit4 mRNA level in the hippocampus of mice with hippocampal stereotactic injection of muscimol or saline. (**C**) qPCR detection of the *Ddit4* mRNA level in the hippocampus of mice with the hippocampal stereotactic injection of MK-801 or saline. (**D**) qPCR detection indicated the *Ddit4* mRNA level in the hippocampus of mice that received sevoflurane exposure with *NMDA* (sevoflurane + *NMDA* group) or saline (sevoflurane group) injection into the hippocampus. Control indicates mice injected with saline in the hippocampus. MK-801 indicates mice injected with MK-801 in the hippocampus. (**E**) Representative pictures showed that DDIT4 level was elevated as shown by western blotting. The data shown are the means ± SDs, *n* = 3. ^ns^*P* > 0.05, ^*^P < 0.05.

*DDIT4*, an encoded protein that regulates development and DNA damage and participates in various pathological processes, was significantly enriched in the regulation of neuron death and positive regulation of cell death (Figure 6A). *NMDAR* and *GABA_A_R* are considered important targets of sevoflurane [36–38]. Therefore, we further explored whether *DDIT4* is regulated by *NMDAR* or *GABA_A_R*. We found that activation of *GABA_A_R* by injection of the *GABA_A_R* agonist muscimol (1.25 μg) into the mouse hippocampus did not cause a significant change in Ddit4 expression (Figure 6B). However, after injection of the *NMDAR* antagonist MK-801 (0.25 μg) into the mouse hippocampus (injection coordinates: AP −2.1 mm, ML 1.5 mm, DV −2.1 mm) by brain stereotactic injection, the mRNA expression of *Ddit4* was increased (Figure 6C).

While using sevoflurane for anesthesia treatment, we injected 0.5 μg *NMDA* into the hippocampus (AP −2.1 mm, ML 1.5 mm, DV −2.1 mm) of mice and found that the increased expression of Ddit4 caused by sevoflurane could be rescued by *NMDA*, indicating that the effect of sevoflurane on the expression of *DDIT4* might occur through the *NMDA* receptor (Figure 6D). The western blot results show that *DDIT4* level was elevated after sevoflurane-treated, but decrease after *NMDA* supplementation (Figure 6E).

## DISCUSSION

As a common perioperative neurological impairment in elderly patients, POCD strongly affects rapid recovery and long-term quality of life and places a heavy burden on patients’ families and society [39]. Neuronal apoptosis induced by sevoflurane is one of the possible factors leading to POCD [10, 40, 41]. Sevoflurane may lead to neuronal death or neuroinflammation to induce cognitive impairment [42]. In this study, we comprehensively analyzed a total of 170 DEGs, 153 upregulated genes and 17 downregulated genes, in the hippocampus of aged rats after sevoflurane anesthesia, and 4 hub genes (*Agt*, *Cdkn1a*, *Ddit4,* and *Rhob*) were critically related to the biological process of cell death. We further confirmed the upregulation of these genes, especially *Ddit4*, in the hippocampus of the aged mice that received 4 hours of sevoflurane anesthesia. *NMDAR*, the core target receptor of sevoflurane, rather than *GABA_A_R*, mediates the sevoflurane regulation of *DDIT4* expression.

We screened the DEGs from the hippocampus of rats, which is closely related to cognitive function [43] and may play an important role in the pathogenesis of POCD [44, 45]. *Agt* encodes angiotensinogen, an angiotensin precursor protein that functions in the renin-angiotensin system (RAS). In addition to the liver, *Agt* is also expressed in the brain. Increasing evidence has shown that the brain RAS plays a key role in Alzheimer's disease, stroke, alcoholism, and depression [46]. Angiotensin regulates iron homeostasis in dopaminergic neurons and microglia through type 1 receptors, thus affecting neurodegenerative diseases such as Parkinson’s disease [47]. The interruption of angiotensinogen synthesis in astrocytes in the rat brain affects the function of the locus coeruleus, which may be responsible for cognitive, behavioural, and sleep disorders [48]. In this study, we found that *Agt* participates in both the positive and negative regulation of neuronal apoptosis. This evidence suggests that the overexpression of *Agt* in the hippocampus of aged rats after sevoflurane anesthesia may lead to dysfunction of the brain RAS system by affecting neuronal apoptosis.

*Cdkn1a* encodes cyclin-dependent kinase inhibitor 1A, which is mainly involved in cell cycle regulation. Several studies have shown that cell cycle-related molecules and pathways play a variety of important roles in influencing neuronal function. In some brain diseases, it is thought that cell cycle arrest may increase the susceptibility to cell death [49]. The failure of cell cycle regulation leads to neuronal dysfunction and cell death, which may be the underlying cause of several neurodegenerative diseases and the ultimate common pathway of other neurodegenerative diseases [50, 51]. Our study confirmed that the *Cdkn1a* gene is enriched in the biological process of positive regulation of cell death, and the overexpression of *Cdkn1a* after sevoflurane treatment may disrupt the normal cell cycle and accelerate neuronal death in the hippocampus. Similarly, the small molecule *GTPase Rhob* encoded by *Rhob* is an important regulator of cytoskeletal tissue and vesicle and membrane receptor transport. Researchers have found that *RHOB* is highly expressed in the hippocampus and may be essential for synaptic plasticity in the hippocampus [52]. Moreover, *Rhob* plays a key role in the apoptotic response, and its deletion affects the apoptotic response of tumor cells to DNA damage [53]. Therefore, both *Cdkn1a* and *Rhob* may be the possible pathological basis of sevoflurane neurotoxicity.

In this study, we found that *Ddit4* is the only key gene enriched in both neuronal death and unidirectional regulation of apoptosis. *Ddit4*, also known as *REDD1* and *RTP801*, encodes proteins that regulate development and DNA damage and participate in a variety of pathological processes. Suppression of *DDIT4* expression decreases cell apoptosis in many kinds of cells [54–56]. Overexpression of *DDIT4* promoted SUNE1 cell proliferation but inhibited apoptosis [57]. Here, we showed that sevoflurane upregulates *DDIT4* expression, which suggests that neuronal apoptosis is induced by sevoflurane neurotoxicity.

The apoptosis-related neuronal death process regulated by sevoflurane leading to cognitive impairment has been recognized. Inhalation of 2% sevoflurane for 5 hours can activate the *NF-κB* signaling pathway and promote neuronal apoptosis and the production of inflammatory factors, thus affecting learning and memory abilities [58]. Activation of the *PI3K/Akt* signaling pathway reduces hippocampal neuronal apoptosis and exerts a protective effect against sevoflurane-induced brain injury in aged rats [59]. We also confirmed that 3% sevoflurane treatment increased the mRNA levels of *Agt*, *Cdkn1a*, *Ddit4*, *Pdgfra*, *Rapgef3*, and *Rhob* in the mouse hippocampus. The expression of *Ddit4* in the hippocampal CA1 region was significantly altered after chronic cerebral hypoperfusion, indicating that it may play an important role in neuronal injury [60]. Inhibition of *DDIT4* could reverse metformin-induced cell cycle arrest and significantly protect against the deleterious effects of the drug on cellular transformation [61]. Inhibition of *DDIT4* expression also exerted a neuroprotective effect after ischemia-reperfusion injury [62]. These results suggest that *DDIT4* may be a key target for intervention in cell apoptosis induced by sevoflurane.

General anesthetics play an anesthetic role mainly by inhibiting the target receptor *NMDAR* and activating *GABA_A_R* to regulate nerve signal transduction and can further induce a wide range of physiological effects through *NMDAR* and *GABA_A_R* to regulate downstream molecular signal pathways [31, 38, 63, 64]. Inhibition of *NMDAR* by MK801 leads to apoptosis of neurons [65, 66]. MK-801 also inhibits proliferation and increases apoptosis in hippocampal neural stem cells [67]. We found that the upregulation of *DDIT4* expression in the hippocampus by sevoflurane can be inhibited through the supplementation of *NMDA* in the hippocampus. The injection of MK-801 into the hippocampus of mice also significantly promoted the expression of *DDIT4*. However, *GABA_A_R* activation did not significantly affect the regulatory effect of sevoflurane on *DDIT4* expression. This finding indicates that sevoflurane regulates the expression of *DDIT4* through *NMDAR* rather than *GABA_A_R*.

However, a limitation in our analysis was that we screened 4 hub genes while we only explored the mechanism of *DDIT4* elevation after sevoflurane inhaling. Mechanisms of the change in the other three genes need to be explored in future experiments. Here we used the antagonist of *NMDAR* MK-801 to determine the *NMDAR* mediating sevoflurane regulation, the gain and loss function of *NMDAR* subunits by RNAi will be performed in the future. In addition, we will further perform the overexpression or knockdown of DDIT4 in the hippocampus to investigate whether Ddit4 is involved in the sevoflurane-induced neuron death.

Our study comprehensively analyzed sevoflurane-regulated DEGs to indicate that *Ddit4* may be a potential target of sevoflurane-induced neuronal apoptosis and determined that the *NMDAR/DDIT4* pathway may be a potential target of sevoflurane neurotoxicity, which provides new possibilities for the prevention and treatment of sevoflurane neurotoxicity.

## MATERIALS AND METHODS

### Microarray data analysis

GSE139220 expression profiles were retrieved and obtained from the NCBI-GEO website (https://www.ncbi.nlm.nih.gov/geo/query/acc.cgi?acc=GSE139220) [68]. The whole transcriptomic data of hippocampal tissue from 3 rats that received 100% oxygen at an identical flow rate for 4 h in an identical chamber and 3 rats that received 2.5% sevoflurane in 100% oxygen for 4 hours in an anesthetizing chamber were included. The raw data were normalized with the rma function by using the oligo package on the R version 4.2.2 platform [69]. The expression data were annotated with the SwissProt database. If the target gene was annotated with two or more probes, the mean value was calculated. Then, the Limma package for the R environment was used to detect the differentially expressed genes (DEGs) in hippocampal tissue between the control group rats and the sevoflurane-treated rats [70]. DEGs were identified based on a *P* value less than 0.05.

### DEG functional enrichment analysis

Gene enrichment analysis of DEGs was performed on the web-based portal Metascape (http://metascape.org/) [71] using the Gene Ontology biological processes and the Kyoto Encyclopedia of Genes and Genomes (KEGG) pathways [34]. The enrichment terms were visualized using the ggplot2 package in R.

### Protein-protein interaction network construction

For all DEGs, a protein-protein interaction (PPI) network was constructed using the STRING database (https://cn.string-db.org/) [72]. Then, the network was visualized on Cytoscape software version 3.9.1, which can be freely downloaded on the website https://cytoscape.org/ and can be used to detect hub genes with the cytoHubba app [73, 74].

### Hub gene selection

To explore the hub genes, we used two screening methods. One is that this gene is involved in multiple biological processes, and the other is that hub genes were screened by 12 algorithms with cytoHubba in Cytoscape, and the genes that were identified by both methods were considered to play a critical role in sevoflurane neurotoxicity. The hub genes enriched in the neuron death process were considered to be involved in neurotoxicity.

### *In vivo* validation

We performed *in vivo* tests to validate the results from the microarray data. The animal experimental protocol was approved by the Standing Committee on Animals at National Cancer Center/National Clinical Research Center for Cancer/Cancer Hospital, Chinese Academy of Medical Sciences and Peking Union Medical College (protocol number: NCC2021A586). Eighteen-month-old C57BL/6J female mice were raised in clean cages under pathogen-free conditions (22–26°C, 12/12 h light/dark cycle) and offered food and water ad libitum. We established a mouse model that received a clinical concentration of 3% sevoflurane (with 40% oxygen and 57% nitrogen) for 4 h of anesthesia (*n* = 3). The temperature was controlled to maintain at 30°C during anesthesia. The control group mice (*n* = 3) were raised under normal rearing conditions.

The *NMDAR* antagonist MK-801 (1 μl, 0.25 μg) (Selleck, USA), the *GABA_A_R* agonist muscimol (1 μl, 1.25 μg) (MedChemExpress, China), *NMDA* (1 μl, 0.5 μg) (Selleck) or 1 μl of saline was injected into the mouse hippocampus (injection coordinates: AP −2.1 mm, ML 1.5 mm, DV −2.1 mm) by brain stereotactic injection.

### Quantitative real-time PCR (qPCR)

Total RNA from the hippocampus was isolated by using RNAiso Plus (TaKaRa, China). cDNA synthesis from mRNA was performed by using the PrimeScript RT Reagent Kit with gDNA Eraser (TaKaRa). Then, the cDNA was used for qPCR detection by using Fast qPCR Mix (TaKaRa). Primers for the qPCR analysis of mRNA are shown as follows:

*Ddit4*-PF: 5′-CAAGGCAAGAGCTGCCATAG-3′, *Ddit4*-PR: 5′-CCGGTACTTAGCGTCAGGG-3′; *Pdgfra*-PF: 5′-AGAGTTACACGTTTGAGCTGTC-3′, *Pdgfra*-PR: 5′-GTCCCTCCACGGTACTCCT-3′; *Rhob*-PF: 5′-GTGCCTGCTGATCGTGTTCA-3′, *Rhob*-PR: 5′-CCGAGAAGCACATAAGGATGAC-3′; *Agt*-PF: 5′-TCTCCTTTACCACAACAAGAGCA-3′, *Agt*-PR: 5′-CTTCTCATTCACAGGGGAGGT-3′; *Cdkn1a*-PF: 5′-CCTGGTGATGTCCGACCTG-3′, *Cdkn1a* -PR: 5′-CCATGAGCGCATCGCAATC-3′; *Rapgef3*-PF: 5′- TCTTACCAGCTAGTGTTCGAGC-3′, *Rapgef3*-PR: 5′-AATGCCGATATAGTCGCAGATG-3′.

### Statistical analysis

Statistical analysis was performed on the R version 4.2.2 platform. The quantitative data are presented as the mean ± SD. The microarray data and *in vivo* PCR validations are displayed with boxplots. Unpaired two-tailed Student’s *t*-test was used to determine significant differences between the two groups. A *P* value less than 0.05 was considered significant.

### Data availability statement

GSE139220 is available from the NCBI-GEO database with the link- https://www.ncbi.nlm.nih.gov/geo/query/acc.cgi?acc=GSE139220. The datasets analyzed during this study are available from the corresponding author upon reasonable request.
